# Decoding Different Reach-and-Grasp Movements Using Noninvasive Electroencephalogram

**DOI:** 10.3389/fnins.2021.684547

**Published:** 2021-09-28

**Authors:** Baoguo Xu, Dalin Zhang, Yong Wang, Leying Deng, Xin Wang, Changcheng Wu, Aiguo Song

**Affiliations:** ^1^The State Key Laboratory of Bioelectronics, Jiangsu Key Lab of Remote Measurement and Control, School of Instrument Science and Engineering, Southeast University, Nanjing, China; ^2^School of Automation Engineering, Nanjing University of Aeronautics and Astronautics, Nanjing, China

**Keywords:** brain-computer interface, electroencephalogram, movement-related cortical potential, reach-and-grasp decoding, neuroprosthesis

## Abstract

Grasping is one of the most indispensable functions of humans. Decoding reach-and-grasp actions from electroencephalograms (EEGs) is of great significance for the realization of intuitive and natural neuroprosthesis control, and the recovery or reconstruction of hand functions of patients with motor disorders. In this paper, we investigated decoding five different reach-and-grasp movements closely related to daily life using movement-related cortical potentials (MRCPs). In the experiment, nine healthy subjects were asked to naturally execute five different reach-and-grasp movements on the designed experimental platform, namely palmar, pinch, push, twist, and plug grasp. A total of 480 trials per subject (80 trials per condition) were recorded. The MRCPs amplitude from low-frequency (0.3–3 Hz) EEG signals were used as decoding features for further offline analysis. Average binary classification accuracy for grasping vs. the no-movement condition peaked at 75.06 ± 6.8%. Peak average accuracy for grasping vs. grasping conditions of 64.95 ± 7.4% could be reached. Grand average peak accuracy of multiclassification for five grasping conditions reached 36.7 ± 6.8% at 1.45 s after the movement onset. The analysis of MRCPs indicated that all the grasping conditions are more pronounced than the no-movement condition, and there are also significant differences between the grasping conditions. These findings clearly proved the feasibility of decoding multiple reach-and-grasp actions from noninvasive EEG signals. This work is significant for the natural and intuitive BCI application, particularly for neuroprosthesis control or developing an active human–machine interaction system, such as rehabilitation robot.

## Introduction

For people with motor disorders, they have normal brain consciousness, but their muscles are damaged, which makes it impossible to implement motion intention. Brain–computer interface (BCI) has created a bridge to transform intentions of people into computer commands *via* deliberate or evoked changes in brain activity (Wolpaw et al., 2002; Schalk et al., 2004; Abiri et al., 2019). Many BCI systems combined with different kinds of applications have been designed for the disabled, such as upper limb prosthesis (Muller-Putz et al., 2019), and other assistive devices (Wolpaw and McFarland, 2004; McFarland et al., 2010; Zhang et al., 2016).

Grasping is an essential skill for humans, which enables us to interact with objects around us. Improving or replacing hand function is the primary demand for tetraplegic persons. For this target group, if surgical rehabilitation fails or is not feasible, there are still some technical solutions that can replace or restore lost hand and arm functions, such as exoskeletons (Prieur-Coloma et al., 2020) or neuroprosthesis (Agashe et al., 2015). Rupp and Gerner (2007) was the first to propose a functional electrical stimulation (FES)-based neuroprosthesis. It rebuilds the function of the muscles by stimulating it with weak periodic electrical pulses and realizes grasping for the first time.

So far, the control strategy of intelligent prostheses, exoskeleton robots, and rehabilitation robots based on noninvasive EEG still relies on the classification of repetitive movement imagination (Wolpaw and McFarland, 2004), movement execution (Jochumsen et al., 2015), externally steady-state evoked potential (SSVEP) (Diez et al., 2013), and event-related potential (ERP) (He et al., 2016). From the psychological point of view of the end user, these control strategies are unnatural and will increase the mental workload because the brain intentions of the users often mismatch the movement of the external device. For instance, imagining the movement of feet controls the opening of the upper-limb neuroprosthesis. In addition, traditional BCI system based on motor imagery has the disadvantage of dissatisfying degrees of control, which cannot satisfy the requirements of high-dimensional control applications.

To solve these problems, it is of great significance to study how the EEG signals, generated by movement execution or attempt movement execution, encode movement information. Previous studies have shown that direction, trajectory, velocity, and acceleration of continuous motor behavior can be decoded from electrocorticographic (ECoG) (Hammer et al., 2013; Bundy et al., 2016). In particular, Bansal et al. (2011) demonstrated that discriminating information for different reach-and-grasp actions and movement detection can be found from invasive ECoG signals below 6 Hz. However, these invasive BCI may suffer from unpredictable risks, like possible postoperative complications and infections. In contrast, many recent works have successfully decoded motor intentions and motor parameters based on noninvasive BCI, especially using movement-related cortical potentials (MRCPs) (Niazi et al., 2011; Lew et al., 2014; López-Larraz et al., 2014; Jochumsen et al., 2015, 2016; Pereira et al., 2017; Iturrate et al., 2018; Zeng et al., 2019; Okorokova et al., 2020).

Recently, a great number of researches have proved that noninvasive EEG signals can be used to decode reach-and-grasp actions. Agashe et al. attempted to decode palmar and lateral grasp from EEG signals. Results indicated that low-frequency time-domain modulation contained discriminative information and can decode grasp kinematics by EEG signals (Agashe et al., 2015). Ofner et al. (2017) decoded six different movements from low-frequency EEG signals, and the multiclass classification performance of executed movements was 55%, while the imagined movement was 27%. Obviously, decoding different movements from the same limb is difficult by using EEG signals. Iturrate et al. (2018) successfully decoded the grasping types and revealed their distinct neural correlates. Studies in healthy subjects showed that palmar, lateral, and pincer grasps could be classified using MRCPs (Schwarz et al., 2017, 2020). Furthermore, Schwarz et al. used the same method to successfully discriminate the three grasping actions of bimanual (Schwarz et al., 2019a).

The above works have some inspiration for our present research. To the best of our knowledge, successfully decoding five different reach-and-grasp movements using MCRPs from noninvasive EEG has not been investigated. Although it is a challenge, it is worth exploring to realize the natural control of BCI application.

In this paper, we aim at decoding five different reach-and-grasp movements closely related to everyday life. They are palmar grasp, pinch grasp, push grasp, twist grasp, and plug grasp. We attempt to study the neural correlation between these five reach-and-grasp actions and decode them by using low-frequency time-domain MRCPs. Assuming that MRCPs has enough distinguishing information to decode them and achieve acceptable decoding performance better than the chance level. An experiment was designed, and our hypothesis was tested in nine healthy volunteers.

## Materials and Methods

### Subjects

Nine healthy subjects (five males, aged 22–25, right-handed) were recruited from Southeast University to participate in the experiment. Every subject had no known history of neurological disease. Before the experiment, subjects were informed about the experimental procedure and signed the informed consent form. This study was approved by the Ethics Committee of Southeast University.

### Experimental Setup and Paradigm

**Figure 1:**
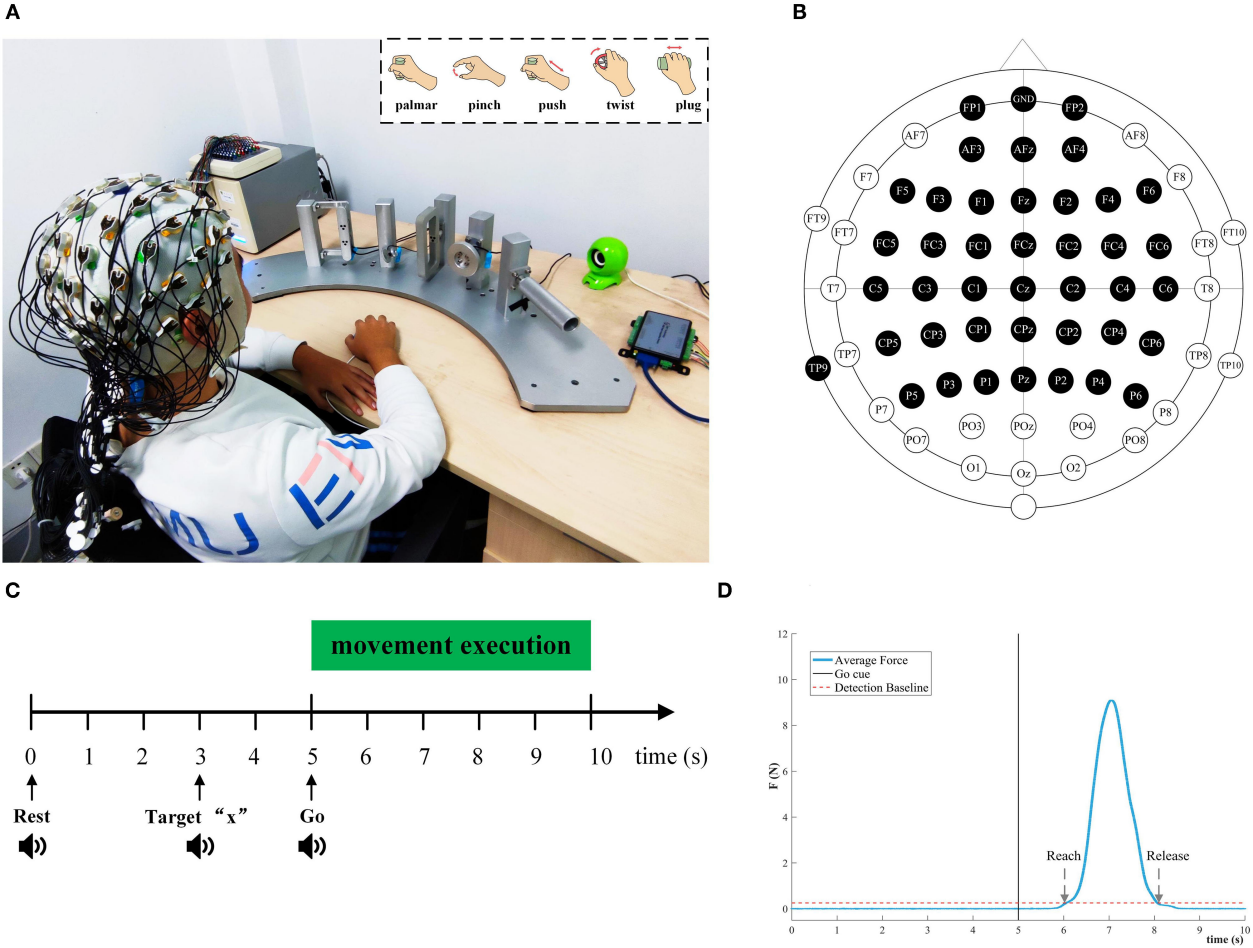
Experimental setup and paradigm for reach-and-grasp tasks. **(A)** An experimental platform for subjects to execute reach-and-grasp actions. The subjects were seated in a comfortable chair. They performed reach-and-grasp actions according to the audio cue displayed by a loudspeaker. Top right: Five reach-and-grasp actions, namely, palmar grasp, pinch grasp, push grasp, twist grasp, and plug grasp. **(B)** The electrodes shown in black were selected in this study. **(C)** The experimental paradigm based on the audio cue. **(D)** The average force curve distribution of the pinch grasping for subject 3, and we calculated the reach and release timepoint of different grasping actions in this way.

Figure 1 depicted the experimental setup and the paradigm. The subject sat on a comfortable chair. Their right hand was relaxed on the push-button on the desk. The position of the push-button is the starting point of the movement. As shown in Figure 1A, five type devices were designed separately based on ergonomics to facilitate reach-and-grasp actions by the subjects. Some mini-nature force sensors were installed on devices and fix the devices on pillars, and the pillars are evenly and equidistantly mounted on a fan-shaped platform. A push-button was placed at the center of the circle of the fan platform to ensure that the subject is equidistant from each object.

During the experiment, five different reach-and-grasp movements were executed by subjects, as shown in the upper right corner of Figure 1A. These reach-and-grasp actions are closely related to daily life and originate from the ipsilateral hand movement area. Additionally, the no-movement condition was recorded to distinguish it from the grasping condition. For the no-movement condition, we instructed the subject to stay in the starting position and avoid any body movement.

The experimental procedure was guided by auditory. Auditory cues were displayed by the computer. The five different grasping actions were mapped to target numbers “1” to “5,” respectively. For the no-movement class, it was mapped to target “6.” Subjects were asked to execute the right-hand grasping task naturally. Figure 1C shows the experimental timing diagram for each trial. Each trial lasts 10 s. At second 0, a beep sounded, and subjects were instructed to put their right hands on the push-button and keep relaxed. Afterward, at second 3, an auditory cue “Target x” indicated the required movement task, and participants were asked to concentrate on it. After 2 s, “Go” cued the subject to execute corresponding reach-and-grasp movements in the way they did in their daily life. In the grasping phase, subjects should give force to the objects as indicated by the arrows in Figure 1A. Since all pillars were fixed, subjects can only perform finger movements rather than wrist movements. The whole reach-and-grasp process, including reaching, grasping, and going back to the starting position, lasts <5 s. For each trial, we had a break for 3 s. Subjects were required to keep the jaw and face muscles relaxed and avoid blinking or swallowing during the experiment for the purpose of reducing artifacts.

In this way, we recorded eight sessions for each subject on the same day. Each session recorded 60 trials (10 trials for each task in random order). After each session, there is a break for 5~10 min. In total, 480 trials were recorded per subject (80 trials per condition).

### Data Acquisition

EEG was measured using a 64-channel active electrode cap (BrainAmp, ActiCap, BrainProducts, Munich, Germany) with international standard 10–20 montage and a SynAmps2 amplifier (Neuroscan Compumedics, USA). We selected 40 active electrodes covering the frontal cortex and the parietal, as shown in Figure 1B. The left mastoid was utilized as the reference and the Fpz channel was chosen as the ground. The impedance of per electrode was kept below 5 K ohm during EEG recording. The EEG sampling frequency was set to 1,000 Hz. A band-pass filter from 0.05 to 100 Hz filtered the EEG signals to attenuate high-frequency band components. To reduce the power line interference, a notch filter at 50 Hz was applied.

A pressure button (rising edge pulse) was adopted to detect the reach-and-grasp movement onset. To record the time point of the reaching, grasping, and releasing hand movements, three flange type force transducers attached to the palmar, plug-and-push grasp devices, a torque transducer fixed to the twist grasp devices, and a miniature I-shaped transducer fastened to the pinch grasp devices were utilized. The analog signals outputted by pressure button and transducers were acquired at 1,000 Hz by using a data acquisition (DAQ) card with USB interface. C++ software was developed to synchronize the DAQ card and EEG acquisition system, and present the auditory cues. Event types of different reach-and-grasp actions were designed and sent to the EEG acquisition system by parallel port communication for marking.

### Movement Onset Detection

During the experiments, subjects naturally executed reach-and-grasp movements guided by auditory cues. Due to the reaction time of subjects, it is unfair to acquire the movement onset using the time point of the auditory cue “Go.” Furthermore, time-locking the movement onset to the auditory cue may produce related evoked potentials, which is inconsistent with our research purpose. Therefore, the information from the pressure button was used to identify the movement onset. The detection procedure of movement onset for each subject was as follows: (1) The time difference between the moment of leaving the button and the “Go” cue is defined as the reaction time (RT) for subjects. We computed the RT of each trial for each subject. (2) Trials with RT > 2 s were found and discarded. (3) The mean and standard deviation of the RT were calculated. We added the mean of RT of each subject to the onset of the authority cue “Go” as the virtual movement onset. For all subjects, totally 94 trials were rejected in this step, including the trials that contained obvious channel noise.

We also investigated the behavior of subjects in the process of performing reach-and-grasp actions. In particular, the time information could be extracted from data provided by the force or torque transducer. As is indicated in Figure 1D, the reach time, release time, and duration of each subject are calculated based on this method. Then, the average duration for each condition was determined.

### Data Preprocessing

#### Rejection Strategies

In this research, we aimed to decode five different reach-and-grasp movements using low-frequency EEG signals, among which EEG was highly affected by ocular and muscular artifacts. To reduce artifacts, particularly from eye artifacts, 33 channels were selected (FP1, FP2, AF3, AF4, AF5, F5, and F6 were excluded since those were highly correlated with ocular) for further analysis. First, the trials with RT exceeding 2 s were discarded. Next, a 40-Hz low-pass filter was utilized to decay high-frequency components from the EEG signals. Finally, statistical parameter method was adopted to reject contaminated trial (Schwarz et al., 2017). Trials whose EEG amplitude dissatisfied the amplitude threshold (amplitude exceeds ±200 μV) were removed. Meanwhile, we rejected these trials where the EEG distribution did not meet the probability statistics (abnormal joint probability or abnormal kurtosis, was >3 times the standard deviation). All the trials that would be removed during signal preprocessing were marked. Averagely, we discarded about 12% of trials owing to artificial artifacts.

#### Signal Preprocessing

First, we removed the trials that were marked by rejection strategies. Then, the EEGLAB toolbox was employed to decompose EEG data by using independent component analysis (ICA) to remove EOG artifacts (Delorme and Makeig, 2004). Specifically, EEG signals was high-pass filtered at 1 Hz, and independent components (ICs) were calculated with the extended infomax ICA implemented in EEGLAB. We then removed the ICs contaminated with eye-related artifacts and projected the remaining ICs back to the original space. Averagely, we rejected 20 ICs per subject by visual inspection. For further analysis, the common average reference (CAR) was implemented to re-referenced EEG data to eliminate the global background activity. Afterward, a zero-phase fourth-order Butterworth bandpass filter from 0.3 to 3 Hz was utilized to extract the low-frequency time-domain components of EEG signals. Finally, the EEG signals were downsampled to 100 Hz. For offline analysis, we epoched our trials 1.5 s before and 2.5 s after the movement onset.

### Movement-Related Cortical Potential

The MRCPs can distinguish the moving body part corresponding to the change in the potential distribution on the scalp. In this research, we were interested to find how the MRCPs generated by movement execution encoded different reach-and-grasp actions. The average value of MRCPs was computed for each channel. Furthermore, the mean overall trials of all subjects and the mean confidence interval (alpha = 0.05) for each condition was calculated. As a representative, the MRCP distribution over the motor cortex was demonstrated. In addition, we performed sample-wise statistical testing based on the nonparametric Wilcoxon rank sum test.

### Feature Extraction and Classification

We are committed to decoding five reach-and-grasp movements by using MRCPs. Hence, the potential amplitude of preprocessing EEG signals was used as features. Features of a single trial were composed of vectors from all electrode channels. Based on the sliding time window, we achieved more features, which is, hopefully, to improve classification performance.

Shrinkage regularized linear discriminant (sLDA) was selected to decode hand-grasping actions (Blankertz et al., 2011). Both binary and multiclass classification were carried out on the preprocessed offline data. The time window [−1, 2.5] s was defined as the time region of interest (tROI), with 0 s corresponding to the movement onset. In this study, the single-trial classification was performed on tROI. The time window size for feature extraction is 500 ms. Specifically, 10 EEG potential amplitudes with the step of 50 ms were chosen as features in this window for each channel. A total of 330 amplitude values were obtained per classification model. The window was slid at 50-ms intervals on the defined tROI for training and testing sLDA model. In this way, 70 individual sLDA classification models were trained and cross-validated within the tROI.

For binary classification, all possible combinations of two conditions were evaluated. For the multiclass classification, we attempted to decode all the grasping excluding the no-movement condition (five conditions in total). The sLDA model of multiclass using a one-vs.-one classification strategy was applied, and five-fold cross-validation of 10 times was performed. Moreover, the peak classification accuracies of subject specific and grand average were reported.

## Results

### Neurophysiological Analysis

**Figure 2:**
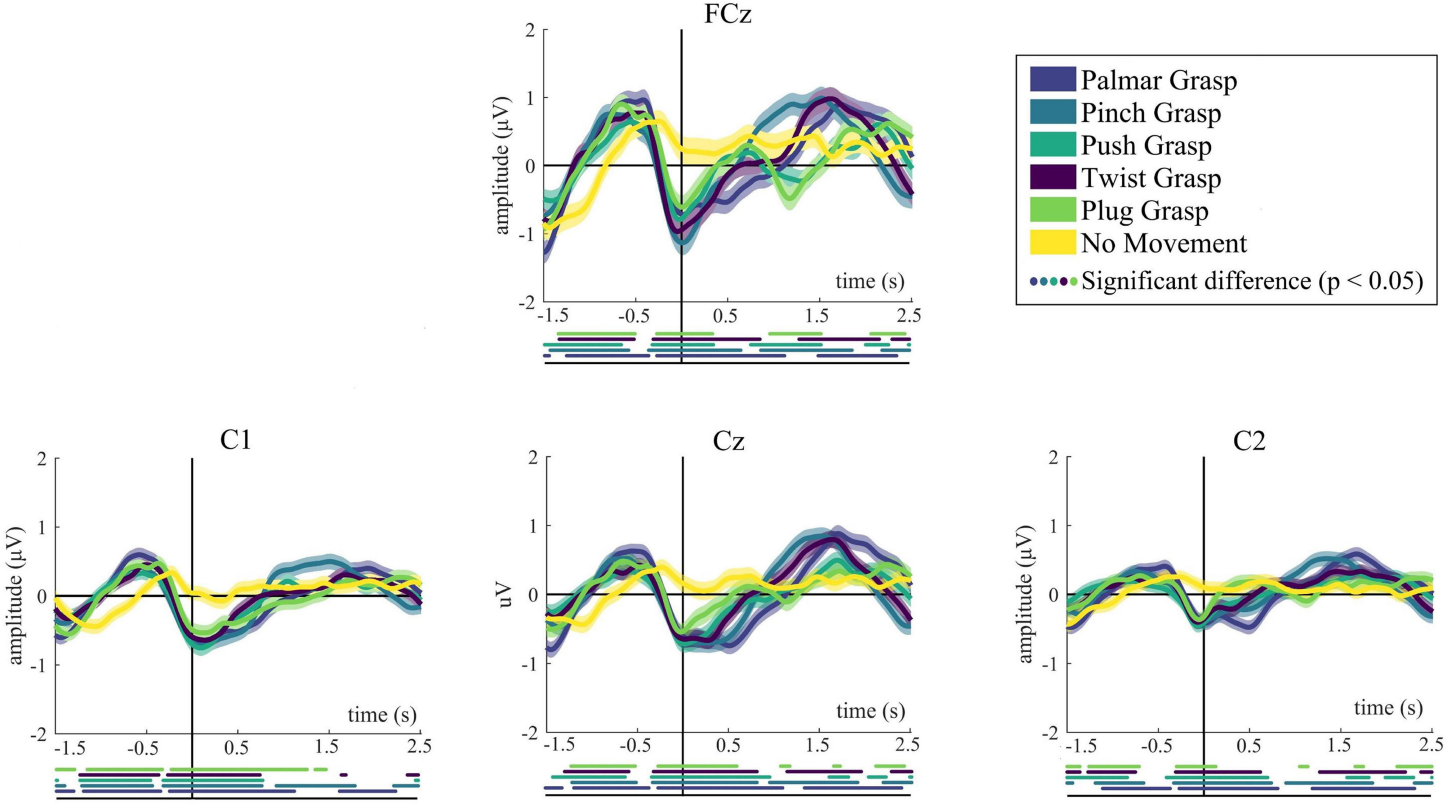
The grand average of all trials of MRCPs for all conditions. Grand average (bold lines) and 95% confidence intervals (shaped areas) for all grasp conditions and the no-movement condition of C1, Cz, and C2. Time = 0 s denotes the starting point of movement. Below the axis, we marked significantly different (*p* < 0.05) timepoints resulting from the Wilcoxon rank sum test between every reach-and-grasp condition and no-movement condition.

Figure 2 demonstrates the grand average MRCPs for five grasping conditions and the no-movement condition. We mainly show the grand-average MRCPs for channel FCz, C1, C2, and Cz on the motor cortex. For the grasping conditions except for the no-movement condition, a strong negative deflection [Bereitschaftspotential, BP (Shibasaki and Hallett, 2006)] was observed. It starts up to around 500 ms before the movement onset and reaches its maximum when the grasping starts. The most significant differences are observed at FCz, Cz, and C1, indicating the contralateral property of the brain. Generally, MRCPs for all grasps conditions are more pronounced than the no-movement condition, especially on FCz and Cz. It is clearly recognizable from Figure 2 that MRCPs of no-movement condition show a similar trend before movement onset, compared with the grasping condition. By contrast, it is completely different after movement execution. Moreover, for all grasping conditions, significant differences with respect to the no-movement condition have emerged 1.5 s before the movement onset.

**Figure 3:**
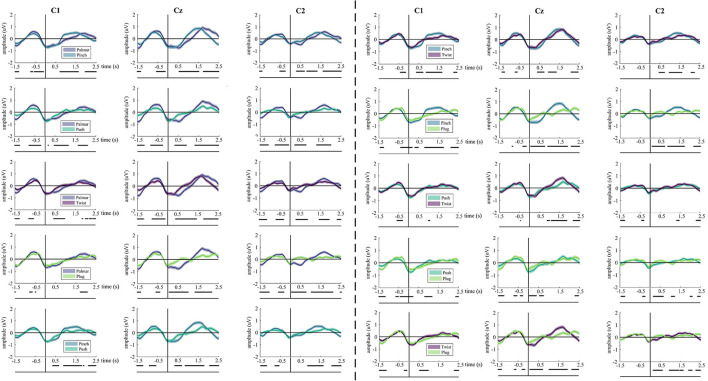
The grand average of all trials of MRCPs for every pair of grasps. Grand average (bold lines) and 95% confidence intervals (shaped areas) for all grasp conditions and the no-movement condition of C1, Cz, and C2. Time = 0 s denotes the starting point of movement. Below the axis, we marked significantly different (*p* < 0.05) timepoints resulting from the Wilcoxon rank sum test for each grasp-vs.-grasp conditions.

The underlying neural differences between grasp conditions were also analyzed. Our study yields 10 binary grasping combinations. Figure 3 illustrates the average MRCPs of all subjects and confidence interval (alpha = 0.05) for each pair of grasp conditions of channel C1, Cz, and C2. In all comparisons between grasp conditions, significant differences can be observed from 0.5 until 2.5 s, especially in the Cz channel. For these combination groups (pinch vs. twist; pinch vs. plug; push vs. twist), there is a similar MRCP morphology prior to movement onset. In addition, these differences are the smallest in the push-vs.-twist comparison. Interestingly, there is almost no difference between push-and-plug combination from 0.5 to 2 s.

**Figure 4:**
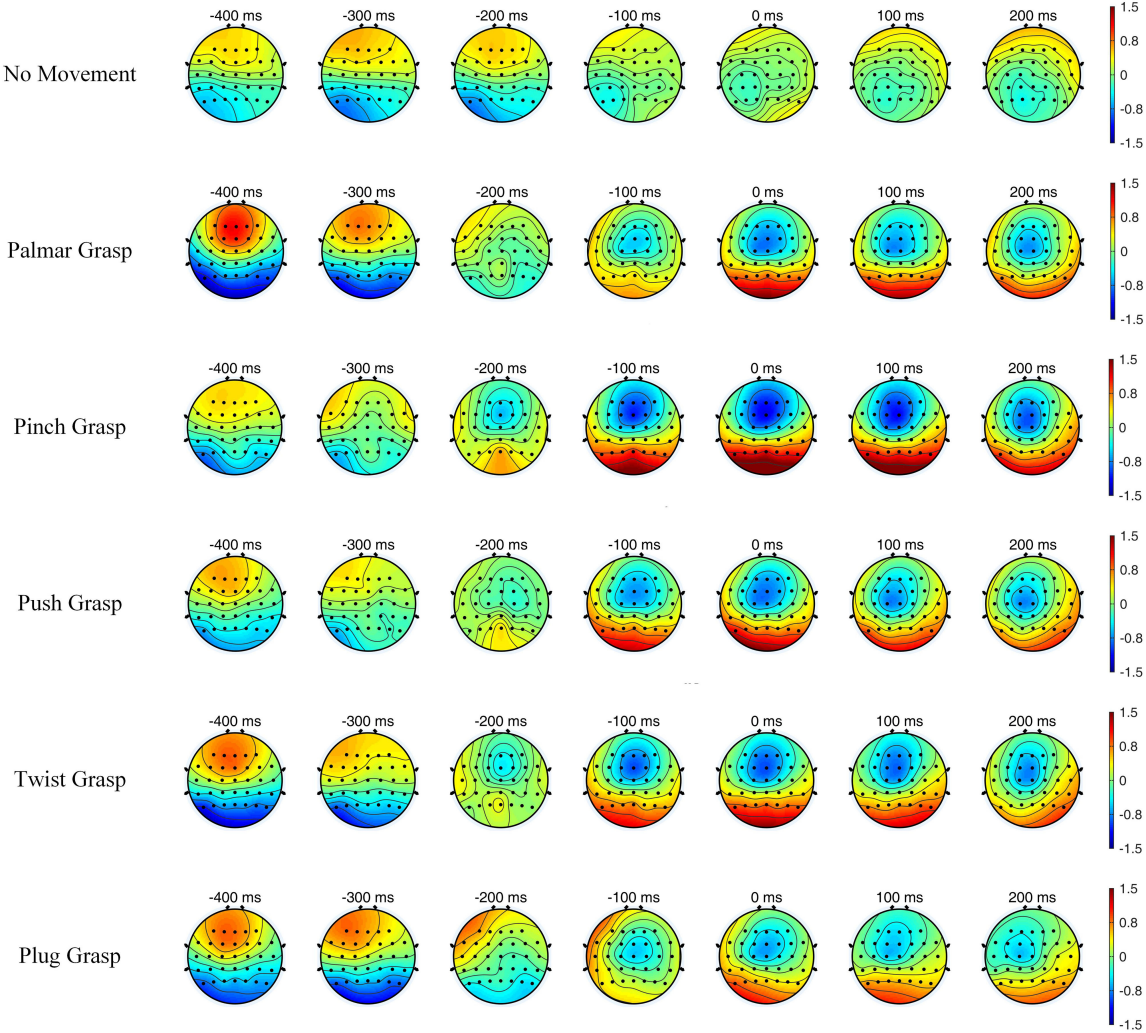
The topographic maps of grand average electroencephalogram (EEG) amplitude over subjects and sessions for all conditions, with time of [−0.4, 0.2] s.

Figure 4 illustrates the topographic maps of grand-average EEG amplitude over subjects, and the time covers 400 ms before and 200 ms after the movement onset. It can be easily seen that the brain region and the degree of activation of grasping conditions are significantly different, compared with the no-movement condition, while different grasping conditions have similarities. Additionally, within the time of [−0.2, 0.2] s, the five reach-and-grasp conditions activated roughly the same brain regions, mainly distributed in the frontal and parietal cortices, while the degrees of activation were different. Such findings indicate that EEG responses induced by different reach-and-grasp conditions are diverse, making it possible to classify different reach-and-grasp movements of the same hand.

### Binary Classification Performance

Single-trial classification was conducted on the preprocessed EEG data. The one-vs.-one classification strategy was implemented for all condition combinations, including grasping-vs.-grasping conditions and grasping vs. the no-movement condition. For binary classification approach, the 500-ms time window was adopted as the feature extraction unit. By this means, the binary classification of all trials was calculated on tROI.

Table 1 demonstrates the subject-specific peak classification performance of grasping vs. the no-movement condition over the tROI, and the occurrence time in comparison with the movement onset. The average peak accuracy of 75.06% was achieved for grasps vs. no-movement, and the subject of S3 got the highest average peak accuracies of 89.63%, while the subject of S6 got the lowest of 68.68%. It is obvious that all the peak accuracies for grasping vs. the no-movement combinations are much higher than the chance level (61.4%, alpha = 0.05, adjusted-Wald interval) (Combrisson and Jerbi, 2015) for all subjects. In addition, there is a similarity in the time when the average peak accuracy occurs for the combinations of grasps vs. no-movement condition except pinch vs. no-movement.

**Table 1:** Subject-specific peak accuracies for grasp vs. no-movement combinations and time of occurrence (s) with respect to the movement onset.

**Grasps vs. no-movement**										
**Subjects**	**Palmar vs. no-movement**		**Pinch vs. no-movement**		**Push vs. no-movement**		**Twist vs. no-movement**		**Plug vs. no-movement**	
	**Acc (%)**	**Time (s)**	**Acc (%)**	**Time (s)**	**Acc (%)**	**Time (s)**	**Acc (%)**	**Time (s)**	**Acc (%)**	**Time (s)**
S1	68.91	1.80	77.22	0.30	70.76	0.25	74.96	0.45	69.65	0.35
S2	66.47	0.40	68.39	−0.05	68.38	0.50	73.89	0.50	71.31	0.35
S3	92.59	1.20	88.89	−0.10	88.89	1.20	85.19	1.20	92.59	1.95
S4	71.00	0.90	75.55	0.55	68.27	1.00	73.18	1.25	76.82	0.40
S5	75.07	2.55	74.29	0.45	65.43	0.55	72.79	2.10	75.21	1.80
S6	67.90	0.05	73.02	0.50	63.28	1.15	72.41	1.85	66.78	0.35
S7	78.25	1.85	73.77	0.10	71.77	1.65	79.87	1.35	71.68	1.40
S8	78.28	0.00	75.98	0.15	75.23	0.05	69.19	−0.15	73.97	0.60
S9	78.57	0.60	78.64	0.10	80.50	1.05	85.43	0.30	77.64	0.60
Mean	75.23 ± 8.0	1.04 ± 0.9	76.19 ± 5.6	0.22 ± 0.22	72.50 ± 8.0	0.82 ± 0.51	76.32 ± 5.8	0.98 ± 0.75	75.07 ± 7.4	0.87 ± 0.66

Table 2 shows the grand average peak performance for the grasping-vs.-grasping condition over the tROI, and the occurrence time relative to the movement onset. It is clear that all grand average peak accuracies for grasping-vs.-grasping combinations are better than the chance level (53.9%, alpha = 0.05) for all subjects. It can be easily observed that the best performance of grasp-vs.-grasp condition is palmar vs. plug, and the classification accuracy reached 68.09% (10.1% standard deviation). In contrast, the lowest is pinch vs. twist, with a classification accuracy of 60.97% (4.6% standard deviation). This is consistent with the similarity of hand movements between pinch grasp and twist grasp in daily life.

**Table 2:** Grand average peak performance for each reach-and-grasp combinations and time of occurrence (s) with respect to the movement onset.

**Task combination**	**Peak accuracy (%)**	**STD (%)**	**Time (s)**
Palmar vs. pinch	65.13	8.3	1.30
Palmar vs. push	61.55	6.2	2.00
Palmar vs. twist	66.53	9.5	1.10
Palmar vs. plug	68.09	10.1	1.25
Pinch vs. push	63.63	7.3	1.45
Pinch vs. twist	60.97	4.6	1.00
Pinch vs. plug	66.95	7.3	0.85
Push vs. twist	65.54	6.1	1.50
Push vs. plug	63.44	6.7	1.00
Twist vs. plug	67.71	8.1	1.65
Mean	64.95	7.4	1.31

### Multiclass Classification Performance

In our research, we focused on distinguishing different reach-and-grasp actions using MRCPs. The multiclass and binary classification approaches were similar in feature extraction, model training, and cross-validation. We also used the 500-ms time window as the feature extraction unit and carried out multiclass classification of all trials on tROI.

**Figure 5:**
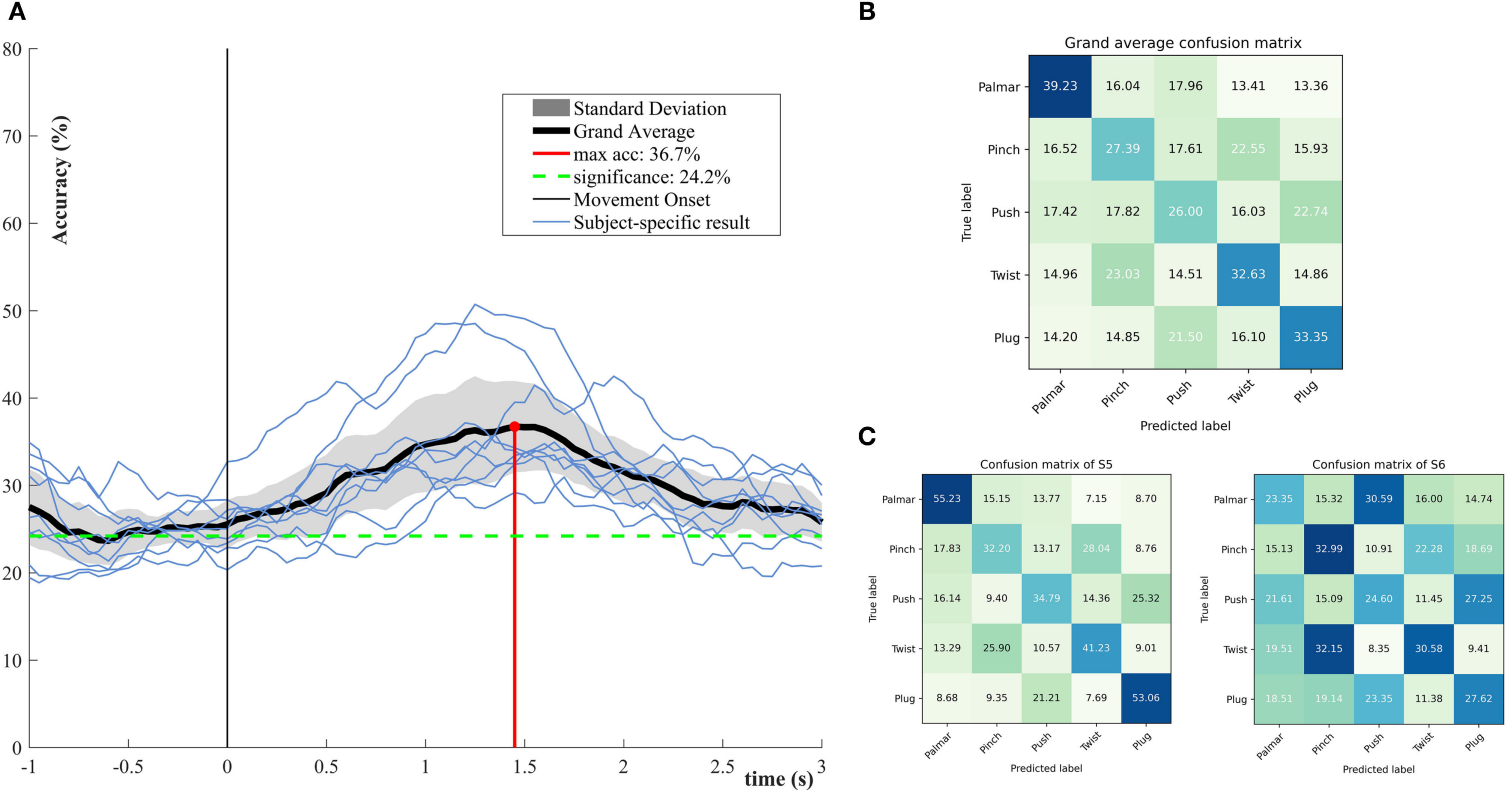
Multiclassification performance over all subjects. **(A)** The grand average classification accuracies including its confidence interval (alpha = 0.05) and subject-specific classification results. **(B)** Grand average row-wise normalized confusion matrix at grand average peak performance. **(C)** Subject-specific row-wise normalized confusion matrix at grand average peak performance, corresponding to the subjects who achieved the highest and lowest performance in **(A)**.

Figure 5 demonstrates the performance of single-trial multiclass decoding of all grasping movements. The multiclass performance of each subject was evaluated on tROI. As shown in Figure 5A, the average of the multiclass performance of all the subjects was computed. The grand average peak accuracy of 36.7% (6.8% standard deviation) is achieved at about 1.45 s. It is better than the chance level (24.2%, alpha = 0.05). Surprisingly, the multiclass classification model superior to the level of chance appeared at 0.5 s before the movement onset. From the results of subject specific, it is obvious that all subjects reached the best classification performance within 1–2 s. Figure 5B illustrates the confusion matrix for all subjects at the time point of peak accuracy. It can be intuitively observed that true positive rates (TPRs) for the grasping conditions range from 26 to 39.23%. The smallest TPR is 26.00% for push grasp, while the highest is 39.23% for palmar grasp. Figure 5C presents confusion matrixes of two subjects who achieve the highest and lowest performance in Figure 5A at the time point of peak accuracy. The TPRs of S5 are higher than that of S6 under almost all grasping conditions. Especially, under palmar and plug grasping conditions, the TPRs of S5 can reach 55.23 and 53.06%, which is higher than S6 with 22 and 16%, respectively.

Table 3 depicts the subject-specific classification results. The classification accuracy of subject 3 gets the highest peak accuracy of 50.72%. Meanwhile, all the subject-specific performances are better than the chance level (29.1%, alpha = 0.05). The average subject-specific peak accuracy of 38.86% (6.8% standard deviation) was achieved. This result is higher than the grand-average peak performance, owing to the time of peak performance varying from subject to subject. In addition, the average time is 1.55 s, which is slightly later than the time of 1.45 s for grand average peak accuracy.

**Table 3:** Multiclassification performance, subject-specific peak accuracies over all subjects, and time of occurrence (s) with respect to the movement onset.

**Subject**	**S1**	**S2**	**S3**	**S4**	**S5**	**S6**	**S7**	**S8**	**S9**	**Mean**
Peak accuracy (%)	33.47	33.99	50.72	34.77	42.49	32.73	37.16	35.85	48.56	38.86 ± 6.8
Time (s)	1.65	1.55	1.25	1.6	1.95	2.25	1.2	1.35	1.15	1.55 ± 0.37

### Behavioral Analysis

**Figure 6:**
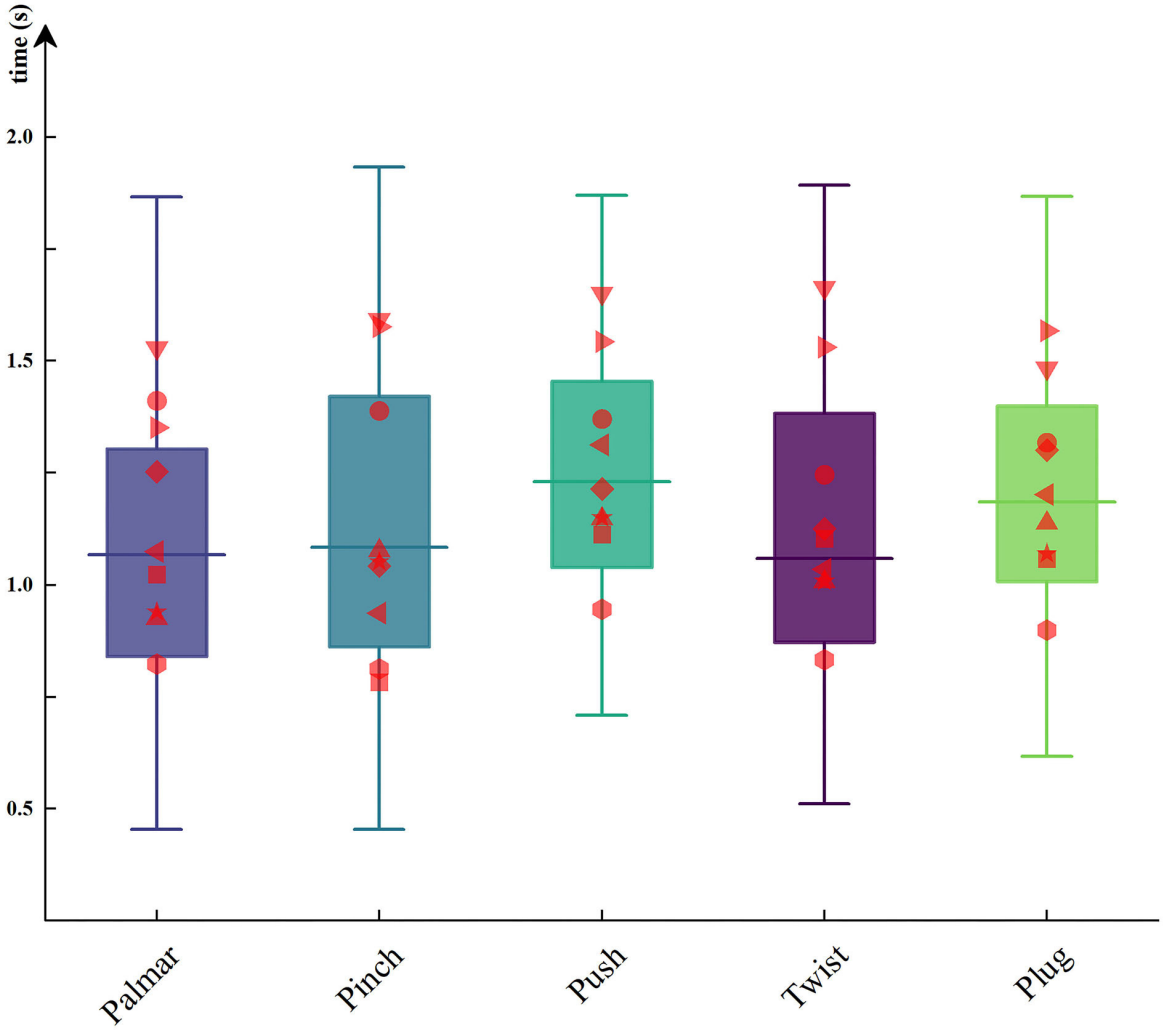
The duration of each hand movement for all subjects. The red dots on the boxplots are subject-specific average duration.

In this paper, we recorded the data of force transducers during the reach-and-grasp movements to obtain the moment of grasp and release. The time when the subject started grasping was calculated, and the results were between 0.98 and 1.18 s after movement onset. Figure 6 shows the duration of each grasping condition for all subjects. The red dots on the boxplots are subject-specific average duration. As can be seen from Figure 6, the duration of the same movement was different between subjects, but the significant difference between the duration of different movements per subject was not observed. By using one-way repeated ANOVA, significant differences of duration were examined. Mauchly's test indicated that the assumption of sphericity was not violated. The reach-and-grasp duration *F* (4, 32 = 8.31, *p* > 0.05) had no significant effect.

## Discussion

In this study, we found that five different reach-and-grasp movements of the right hand could be successfully decoded using noninvasive EEG signals. The underlying neural correlation for all grasping and the no-movement condition was revealed. First, for binary classification, average performance for grasping vs. the no-movement peaked at 75.06 ± 6.8%. Second, for grasping-vs.-grasping conditions, a peak average performance of 64.95 ± 7.4% could be reached. Finally, for multiclass classification, five reach-and-grasp actions were successfully decoded and grand-average peak performance reached 36.7 ± 6.6%. Better-than-chance classification accuracies were achieved for both binary classification and multiclass classification approach. Our findings provide the possibility and potential for intuitive and natural neuroprosthesis control and also contribute to future research on decoding hand movement information.

### Movement-Related Cortical Potentials

Results of the MRCPs shows that a typical negative peak around movement onset, which is in accordance with the previous studies (Schwarz et al., 2017, 2019b). A negative shift occurs about 500 ms prior to the movement onset and reaches the maximum at movement onset, which associates with the BP. The distribution of MRCPs is most noticeable in the central motor cortex for FCz and Cz, and C1 (contralateral side) is more noticeable than on C2 (ipsilateral side). At around 0.2–0.5 s, all grasping conditions demonstrate a weak second positive rebound except push-and-plug grasp conditions in which there is a large second positive rebound at about 1.2 s.

All grasping conditions achieved significant differences over the whole tROI. For the grand-average MRCPs of grasp-vs.-grasp conditions, there is almost no difference between these pairs (push vs. plug; push vs. twist), and thus, the classification accuracy was low. A reasonable explanation is that similarity between these action pairs is higher than other actions. By contrast, it can be observed that significant differences have emerged from 0 to 2.5 s within the remaining reach-and-grasp combinations.

### Single-Trial Classification

For the binary classification, all possible condition combinations were studied. First, the results for grasping vs. the no-movement conditions were computed, and classification results with average peak accuracies over 75.06% were obtained. Peak accuracies reached within 0.5–1.5 s, while pinching vs. no-movement was at 0.2 s. The significance level of classification level reached before movement onset within movement vs. no-movement conditions. We found that movement intention can be detected in advance for all subjects. This is particularly important for BCI control because it can realize the natural transformation from the idle to control. Second, for all movement vs. movement task combination, classification performance was better than chance level. The best performance of the task combination is about 10% better than the lowest. Therefore, for future applications, a subset of classes that can work best for BCI users should be selected.

Consistent with the study of Agashe et al. (2015), we also ignored the no-movement condition for multiclassification. A peak accuracy of 36.7% was reached at 1.5 s behind the movement onset. For the multiclass scenario, better-than-chance decoding accuracy could be achieved 500 ms prior to the movement onset. This means that detecting movement intention of multiple grasping is feasible, which is fundamental for achieving natural and asynchronous BCI control. By analyzing the multiclass-based confusion matrices, we found that grasping-vs.-grasping conditions contributed unevenly to the overall performance. The TPR of pinch grasp is lowest, indicating that it is not sufficiently distinguishable from the other grasping conditions, which is consistent with the binary classification results.

The time for peak accuracy of binary and multiclass classification for all reach-and-grasp conditions was similar. The binary classification was at 1.31 s, while multiclass classification was at 1.45 s, which indicated that peak accuracy occurs during the grasping itself and is in accordance with the previous studies (Schwarz et al., 2017). Unfortunately, due to differences in the experimental setup and paradigm, it is difficult to compare directly other similar studies.

### Limitations and Future Work

Our findings make an important contribution to the field of controlling neuroprosthesis naturally and intuitively and improving the dimensionality of the BCI control system. However, our study still has limitations.

First of all, we use an audio-based protocol, and it will elicit the corresponding EEG, which might mask the MRCPs. The previous study showed that there was less pronounced negativity in MRCP in the movement preparation phase for cued movements compared with self-paced movement (Savić et al., 2014). In addition, Scheel et al. found that the negative peak of MRCP was significantly higher during movement execution in the auditory paradigm than in visual paradigm (Scheel et al., 2015). In the future, we will conduct in-depth research on the impact of audio or visual cues on MRCPs.

Next, we cannot completely rule out the influence of direction on the decoding before grasping phase, since the position of five devices in our experiment setup was fixed. The study of Kim et al. showed that direction and distance had the most contribution to the movement, while there was no significant difference observed for position (Kim et al., 2019). In our experiment, five devices were arranged in a fan shape to ensure equal distance. In the follow-up study, we will improve our experiment paradigm by rearranging the devices in clockwise order after each session.

Besides, we did not consider the influence of object proprieties for interpreting EEG activity. In the latest studies, Sburlea et al. (2021) found that the information about grasp types and object properties encoded in MRCP was not represented in isolation through channels in one brain region; instead, channels covering different brain regions processed both types of information at several stages of movement. In our study, the multiclass classification accuracy reached chance level starting in the planning phase, which was in line with their results. However, our study did not keep object proprieties (shape and size) consistent. Therefore, the influence of object proprieties for interpreting EEG activity will be considered in our future work.

In addition, we did not perform our study on the spinal cord injured (SCI) subjects. In general, there are differences in cortical activation between healthy subjects and patients who have neural lesions (e.g., stroke). For future works, it urgently needs to verify our findings on SCI subjects, although it is challenging. In detail, we desire to confirm whether the decoding performance of the motor imagery and SCI group is closer to the performance reported in our work.

Last, the presented analysis is focused mostly on the amplitude of the signal; more sophisticated analysis may yield higher decoding accuracy and satisfy the requirements of BCI applications. Previous studies have shown that wavelet transform time-frequency image and EEG source imaging can achieve excellent performance in motor imagery classification (Xu et al., 2019; Hou et al., 2020). Besides, Schwarz et al. combines the MRCP amplitude and the features based on SMR modulations, and the decoding accuracy is 10% higher than using the simple time-domain features (Schwarz et al., 2019b), which gives us inspiration to combine frequency and time-domain features in the further study. Additionally, deep learning architectures are widely used in EEG signal analysis to boost classification accuracy, especially in MI tasks (Lawhern et al., 2018; Craik et al., 2019). Further studies are possible to combine hybrid features and deep learning algorithms to boost accuracy and evaluate the decoding performance for a larger population.

## Conclusion

In this paper, we have demonstrated the feasibility of decoding five executed reach-and-grasp actions from the same hand by using MRCPs. The neurophysiological analysis has revealed that MRCPs for all grasp conditions are more pronounced than the no-movement condition, and MRCPs between grasp conditions show significant differences. Moreover, single-trial classification accuracies for binary and multiclass are significantly better than the chance level. These findings are of great importance for the natural and intuitive control of BCI, particularly for neuroprosthesis or rehabilitation robots.

## Data Availability Statement

The raw data supporting the conclusions of this article will be made available by the authors, without undue reservation.

## Ethics Statement

The studies involving human participants were reviewed and approved by Ethics Committee of Southeast University. The patients/participants provided their written informed consent to participate in this study.

## Author Contributions

BX and DZ designed the study, analyzed the data, and wrote the manuscript. DZ and YW set up the experiment platform. LD and XW performed the experiment. CW and AS were involved in the critical revision of the manuscript. All the authors read and approved the final manuscript.

## Funding

This work was partially supported by grants from the National Key Technologies Research and Development Program (No. 2019YFC0119303), the Basic Research Project of Leading Technology of Jiangsu Province (No. BK20192004), and National Natural Science Foundation of China (Nos. 61673114, 91648206, and 61803201).

## Conflict of Interest

The authors declare that the research was conducted in the absence of any commercial or financial relationships that could be construed as a potential conflict of interest.

## Publisher's Note

All claims expressed in this article are solely those of the authors and do not necessarily represent those of their affiliated organizations, or those of the publisher, the editors and the reviewers. Any product that may be evaluated in this article, or claim that may be made by its manufacturer, is not guaranteed or endorsed by the publisher.
